# Scale-dependent biodiversity–biomass relationships vary among subtropical forest community types

**DOI:** 10.3389/fpls.2026.1869844

**Published:** 2026-07-15

**Authors:** Theint Theint Soe, Boliang Wei, Wenjie Zhou, Zhongjie Yang, Zhibin Mao, Zhonghan Wang, Lei Zhong, Donghao Wu, Jinliang Liu, Mingjian Yu

**Affiliations:** 1 State Key Laboratory for Vegetation Structure, Function and Construction (VegLab), MOE Key Laboratory of Biosystems Homeostasis and Protection, College of Life Sciences, Zhejiang University, Hangzhou, China; 2 Zhejiang Wuyanling National Reserve Management Bureau, Wenzhou, China; 3 College of Life and Environmental Science, Wenzhou University, Wenzhou, China

**Keywords:** aboveground biomass, alpha diversity, beta diversity, biodiversity–biomass relationships, scale–dependent effects, subtropical broad–leaved forests

## Abstract

Exploring how biodiversity influences forest biomass is critical to biodiversity–ecosystem functioning (BEF) research. However, the combined effects of species diversity and compositional heterogeneity among communities and spatial scale on aboveground biomass (AGB) remain insufficiently resolved. We quantified species alpha diversity, beta diversity, and AGB in 15 one-hectare permanent forest plots representing five forest community types across three spatial scales (10 m, 20 m, 50 m) in eastern China, then analyzed the relationships between diversity and AGB. The results showed that positive relationships between alpha diversity and aboveground biomass were consistently observed in evergreen broad-leaved forests and low-elevation evergreen and deciduous broad-leaved mixed forests, and these relationships generally became stronger with increasing spatial scale in these forest types. In contrast, high-elevation mixed forests exhibited weak or negative associations between alpha diversity and biomass. For beta diversity, significant positive associations with biomass differences were detected at the 10 m scale across all forest communities. At 20 m scale, these relationships remained predominantly positive but were less uniform among community types, with some variation in direction and strength. At 50 m scale, the relationships weakened and became more variable among communities, showing a mixture of positive, negative, and non-significant patterns depending on forest type. Collectively, our findings highlight that incorporating spatial scale and forest type is essential for interpreting biodiversity–biomass relationships, and conserving both local species diversity and compositional heterogeneity is very important for sustaining forest productivity, carbon storage, and ecosystem stability.

## 1 Introduction

Understanding the relationship between plant diversity and forest biomass is one of the key topics of biodiversity–ecosystem functioning (BEF) research (Gamfeldt et al., 2008; Germany et al., 2021). Biodiversity can affect biomass through mechanisms such as niche complementarity, facilitation, and selection effects associated with dominant species (Tilman et al., 1997; Loreau and Hector, 2001). Although increasing evidence indicates that biodiversity–biomass relationships are influenced by community composition, environmental conditions, and spatial scale (Jucker et al., 2014; Ali et al., 2016), it remains unclear whether the effects of alpha diversity and beta diversity on aboveground biomass are consistent across forest community types and spatial scales. While previous studies have often reported scale-dependent or even conflicting patterns in biodiversity–biomass relationships, fewer studies have explicitly examined how differences in forest communities along environmental gradients, such as elevation, influence these scaling relationships when multiple components of biodiversity are considered simultaneously. In particular, integrated analyses that compare the responses of alpha and beta diversity across contrasting subtropical forest types and multiple spatial resolutions are still limited, leaving unresolved how community composition may modify scale-dependent biodiversity–ecosystem functioning relationships (Chen et al., 2022; Xu et al., 2022).

Alpha diversity describes the diversity of species coexisting within a local community and is widely recognized as an important driver of ecosystem functioning and aboveground biomass (AGB). At fine spatial grains, alpha diversity is strongly associated with neighborhood interactions among species, where niche partitioning, facilitation, and competitive dynamics can improve resource utilization and productivity (Tilman et al., 1997; Loreau and Hector, 2001; Cardinale et al., 2007). As a result, alpha diversity largely reflects local ecological processes that contribute to biomass accumulation. By comparison, beta diversity characterizes variation in species composition among communities across spatial and environmental gradients (Whittaker, 1960; Socolar et al., 2016). In forest ecosystems, beta diversity reflects compositional differences that may arise from habitat heterogeneity and environmental filtering. Consequently, beta diversity is often linked to broader-scale ecological processes, including species replacement and habitat differentiation, which can influence spatial patterns of biomass and ecosystem functioning (Loreau et al., 2003; Mori et al., 2018).

In addition, biodiversity–biomass relationships may vary across spatial scales. Previous BEF studies have demonstrated that diversity and biomass are predominantly regulated by neighborhood-scale interactions, local species coexistence, competition, and niche complementarity. These fine-scale mechanisms are therefore essential for disentangling how tree interactions shape forest productivity, particularly in species-rich subtropical forests (Tilman et al., 1997; Chisholm et al., 2013; Yuan et al., 2020; Mori et al., 2021). In contrast, studies conducted at broader spatial extents have emphasized environmental heterogeneity, habitat filtering, species turnover, and landscape-scale community assembly processes shaping biodiversity–ecosystem functioning relationships (Condit et al., 2002; Legendre et al., 2009; Wang et al., 2017; Luo et al., 2024). Because local ecological processes are fundamental to understanding tree interactions and biomass accumulation in subtropical forests, we focused on the 10 m – 50 m spatial scales in this study to evaluate how biodiversity–biomass relationships vary among forest community types and across spatial grains.

**Figure 1:**
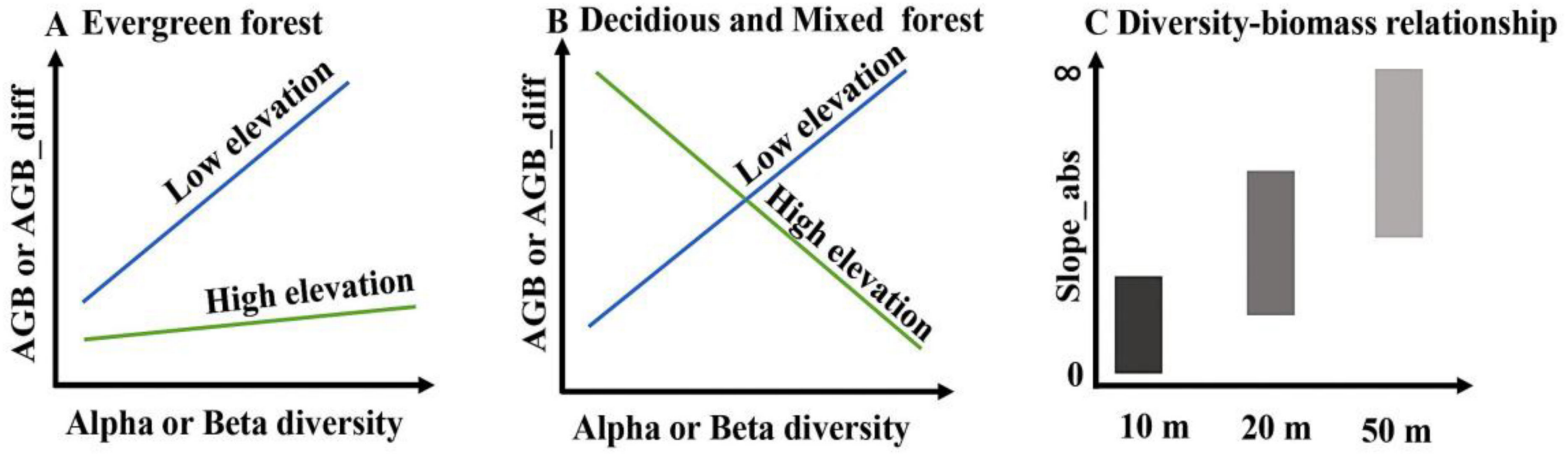
Conceptual framework showing how biodiversity relates to aboveground biomass (AGB) across forest types and spatial scales. Conceptual relationships between alpha or beta diversity and AGB or AGB_diff in evergreen forests **(A)** and in deciduous and mixed forests **(B)** at different elevations, illustrating how biodiversity–biomass relationships may vary with environmental conditions and biotic interactions. Panel **(C)** conceptually illustrates an increase in the absolute strength of these relationships across spatial scales. In panels **(A, B)**, blue lines indicate low–elevation forests, and green lines indicate high–elevation forests. In panel **(C)**, taller bars indicate larger absolute slope values. These panels represent conceptual hypotheses rather than explicit quantitative predictions.

Building on biodiversity–ecosystem functioning (BEF) theory and community assembly frameworks, we developed hypotheses to explore how relationships between biodiversity and aboveground biomass (AGB) vary across forest types, elevational gradients, and spatial scales (**Figure 1**). Evidence from earlier research suggests that greater species richness is associated with increased biomass production, driven by niche complementarity, facilitative interactions, and dominance effects within communities (Tilman et al., 1997; Loreau and Hector, 2001; Cardinale et al., 2007; Liang et al., 2016). However, the strength and direction of biodiversity–AGB relationships may differ among forest communities because evergreen and deciduous species exhibit contrasting ecological strategies and responses to environmental stress.

In evergreen forests, biodiversity was expected to contribute consistently to biomass accumulation across elevations. Compared with deciduous species, evergreen trees are often characterized by resource-conservative ecological strategies, including extended leaf longevity, greater tolerance of shaded environments, slower rates of resource acquisition, and enhanced resistance to environmental stress. These characteristics may contribute to more stable biomass accumulation and sustained species complementarity across elevational gradients (Aerts, 1995; Reich, 2014). Therefore, we hypothesized that evergreen forests would exhibit relatively consistent biodiversity–AGB relationships along the elevational gradient. Meanwhile, given that low-elevation habitats generally feature more favorable environmental conditions than high elevations, the correlation strength is expected to be stronger at lower altitudes (**Figure 1A**).

In contrast, deciduous and mixed forests were expected to exhibit more variable biodiversity–AGB relationships between low and high elevations (**Figure 1B**). Under favorable low-elevation conditions, higher species diversity may promote biomass accumulation through complementary resource use and facilitative interactions among coexisting species (Loreau and Hector, 2001; Tilman et al., 2014). However, at high elevations, environmental stressors such as lower temperatures, shorter growing seasons, and nutrient limitations may increase the importance of environmental filtering and reduce the relative contribution of species complementarity. Consequently, biodiversity–AGB relationships in deciduous and mixed forests were expected to become weaker or less consistent under more stressful conditions.

Furthermore, we hypothesized that biodiversity–AGB relationships would become weak with spatial scale because broader spatial grains capture a wider range of species interactions and spatial variation in community composition (**Figure 1C**). Fine spatial scales mainly reflect local neighborhood interactions and competitive processes, whereas larger spatial scales increasingly incorporate broader patterns of species turnover and habitat variability that may influence biomass accumulation through spatial complementarity among species (Loreau et al., 2003; Isbell et al., 2017).

To fill the knowledge gap regarding how biodiversity–biomass relationships vary across community types and spatial scales, we quantified alpha diversity, beta diversity, and AGB in 15 one-hectare permanent forest plots representing five forest community types across three spatial scales. We propose a conceptual framework (**Figure 1**) linking biodiversity–biomass relationships to environmental gradients and spatial scale through shifts in dominant ecological processes, from local species interactions at finer scales to broader compositional heterogeneity at larger spatial extents. Specifically, we address the following questions: (1) How do alpha diversity and beta diversity vary across forest community types and spatial scales? (2) Do these diversity components exhibit varying scale-dependent relationships with aboveground biomass across contrasting forest community types?

## 2 Materials and methods

### 2.1 Study site, plot establishment, and data collection

**Figure 2:**
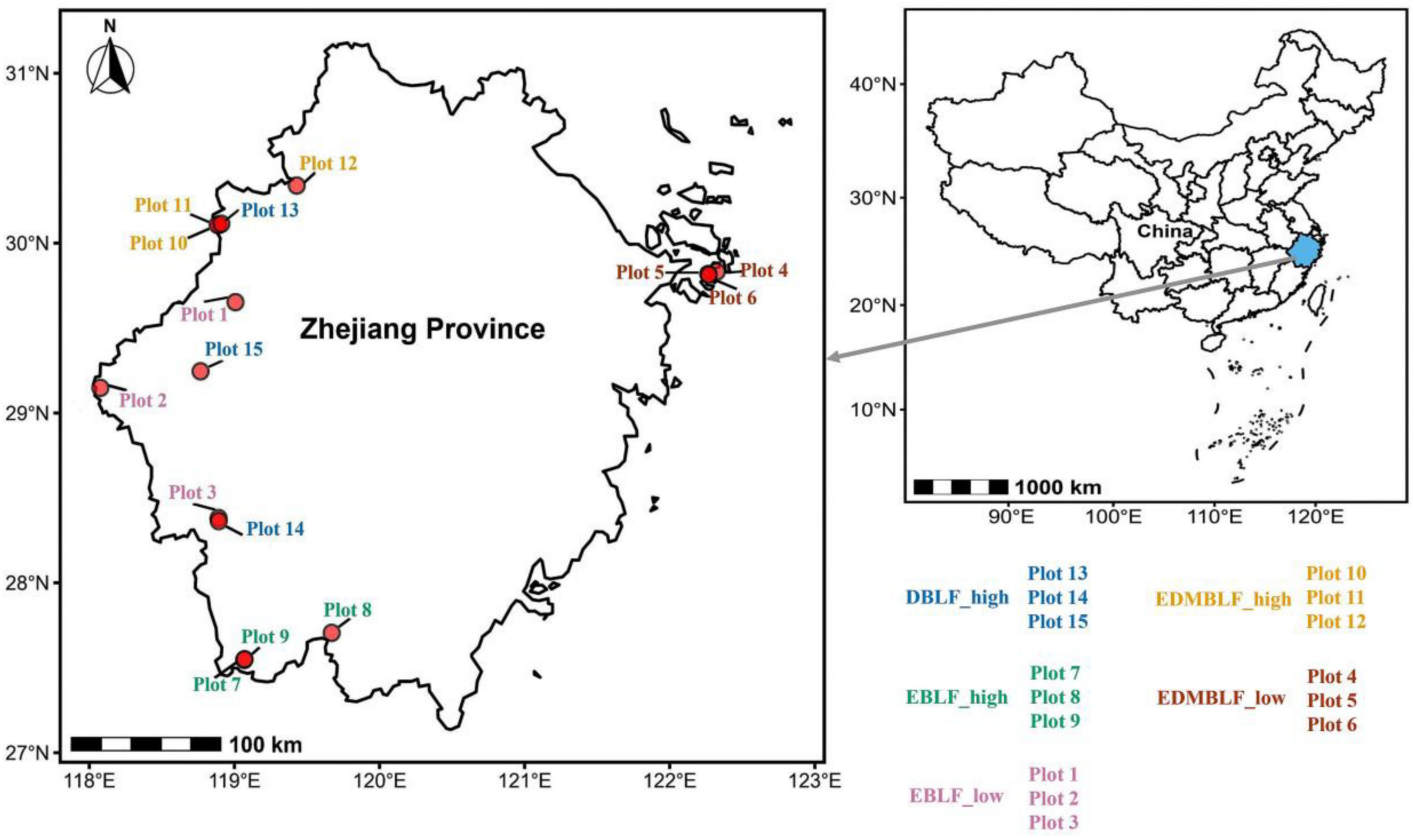
Study area showing the location of Zhejiang Province in China and the spatial distribution of study sites.

The study was conducted in Zhejiang Province, eastern China (27.03°–31.18°N, 118.02°–123.17°E; **Figure 2**), a region dominated by mountainous and hilly terrain with over 60% forest coverage. The climate is a monsoon in the middle subtropics, described as hot and wet in summers and cold in dry winters, with prominent seasonal variations. The average annual temperature in Zhejiang ranges from 15 °C to 18 °C, with annual precipitation varying between 980 mm and 2000 mm. In western Zhejiang, elevations range from 100 to 1,500 m in low hills and plains, while the highest peak, Huangmao Peak in the Donggong Mountain Range, reaches 1,929 m above sea level.

A total of 15 1–ha permanent forest plots were established according to standard CTFS–ForestGEO protocols (Anderson-Teixeira et al., 2015). All trees with a diameter at breast height (DBH) ≥ 1 cm and a height ≥ 1.3 m were measured, tagged, and mapped. DBH was measured with a diameter tape, and height was determined using a measuring rod. Species identification and classification followed the taxonomic criteria outlined in the Flora of China (http://www.iplant.cn/foc), the Flora of Zhejiang (New Edition) (Editorial Committee of Flora of Zhejiang, 2021).

The plots were classified into five forest community types based on forest composition and elevation, including low– and high–elevation evergreen broad–leaved forest (EBLF_low and EBLF_high), low– and high–elevation evergreen–deciduous mixed broad–leaved forest (EDMBLF_low and EDMBLF_high), and high–elevation deciduous broad–leaved forest (DBLF_high). To support vegetation classification, we used a digital elevation model (DEM) to extract the 800 m contour line, which corresponds to the boundary between warm and temperate coniferous forest zones in the mountainous areas of Zhejiang. This elevation threshold was further applied to distinguish mountain and low-mountain vegetation types within the four-level classification framework (Hu et al., 2014; Zhejiang Ecological and Environmental Restoration Technology Association, 2025). Accordingly, this study adopted 800 m as the dividing line between low- and high-elevation sites in Zhejiang Province, with low-elevation sites defined as those below 800 m and high-elevation sites as those above 800 m. For each community type, three plots were selected at comparable elevations to represent similar elevational conditions. Detailed information on plot distribution is provided in **Supplementary Table 1**. The geographic coordinates of each plot were recorded using a global positioning system, and elevations ranged from 61 to 1,544 m above sea level.

### 2.2 Diversity matrices

Alpha species diversity was quantified using Shannon–Wiener’s diversity index (*H′*) (Shannon and Weaver, 1963). Pairwise Bray–Curtis dissimilarity (dBC) was calculated from species abundance data (x_ij_) following Baselga (2013) to quantify compositional differences between plots.

### 2.3 Estimation of aboveground biomass

Aboveground biomass (AGB) was estimated non–destructively from tree DBH and height measurements, following Yilma and Derero (2020). Individual tree biomass was calculated using species–specific allometric equations when available, with a generalized model applied for species lacking specific equations. Tree–level biomass values were summed to obtain plot–level AGB estimates. Full details of the allometric equations and their sources are provided in **Supplementary Material II**.

### 2.4 Calculation of AGB differences for beta diversity assessment

The absolute difference in AGB (AGB_diff) for each subplot sampling unit pair was calculated as the absolute value derived by subtracting the biomass of one sampling unit from the other (Baselga, 2013). These values were then used to assess the relationship between AGB differences and beta diversity (Bray−Curtis dissimilarity) across different spatial scales.

### 2.5 Statistical analysis

To examine scale–dependent biodiversity patterns, species diversity and aboveground biomass (AGB) were analyzed at three spatial scales (10 m × 10 m, 20 m × 20 m, and 50 m × 50 m) across different forest types. Each 100 m × 100 m permanent plot was initially subdivided into contiguous 10 m × 10 m subplots as the basic sampling units. Larger spatial grains were subsequently generated by aggregating neighboring subplots into nested 20 m × 20 m and 50 m × 50 m quadrats rather than applying random sampling procedures (Chave et al., 2014).

Alpha diversity was calculated for each subplot at the corresponding spatial scale, whereas beta diversity was quantified using Bray–Curtis dissimilarity based on species abundance data from pairwise comparisons among subplots. Biodiversity indices were compared across all sampled sites to assess differences among communities. Non–parametric Kruskal–Wallis tests were applied to evaluate variation in species diversity and beta diversity indices, with pairwise comparisons conducted using Dunn’s test and Bonferroni correction (Haselberger et al., 2021). To assess variability within each community type, additional plot–level comparisons were performed, with results summarized in **Supplementary Figures 1** and **S2**.

Prior to model fitting, Moran’s I statistics were used to test for spatial autocorrelation in aboveground biomass (AGB) and diversity variables across all spatial scales (**Supplementary Tables 10**, **S11**). Accordingly, spatial lag regression models were applied to analyze the relationships between AGB and alpha diversity, as well as between aboveground biomass differences (AGB_diff) and Bray–Curtis dissimilarity, separately for each community type and spatial scale to account for spatial dependence among neighboring subplots (Anselin, 1988). Because beta diversity and biomass differences were calculated from pairwise comparisons among spatially connected subplots, observations were not fully independent. AGB and AGB_diff were log-transformed to meet model assumptions, and all explanatory variables were standardized using Z-score standardization prior to model fitting. Spatial neighborhood relationships were defined using k-nearest neighbor matrices based on subplot coordinates. Residual spatial autocorrelation of the fitted models was further evaluated using Moran’s I statistics (Moran, 1950; Bivand et al., 2013) (**Supplementary Tables 12**, **S13**). To further examine whether the biodiversity–biomass relationship differed between elevation zones (high vs. low elevation), spatial lag interaction models were fitted by including elevation class and its interaction with biodiversity metrics as fixed effects (**Supplementary Figures 4**, **S5**). All analyses were performed in R version 4.3.3 (R Core Team, 2024).

## 3 Results

### 3.1 Variation in alpha and beta diversity and aboveground biomass across spatial scales and community types

Both alpha and beta diversity among forest community types showed scale–dependent differences (**Supplementary Tables 2**–**S5**). For alpha diversity, highly significant differences were detected at 10 m, 20 m, and 50 m (*p <* 0.001), with the largest χ² values observed at 10 m and smaller values at broader spatial scales (**Supplementary Tables 2**, **S3**). Similarly, beta diversity measured by Bray–Curtis dissimilarity showed the largest differences at 10 m, remained significant at 20 m, and was lower at 50 m (**Supplementary Tables 4**, **S5**).

**Figure 3:**
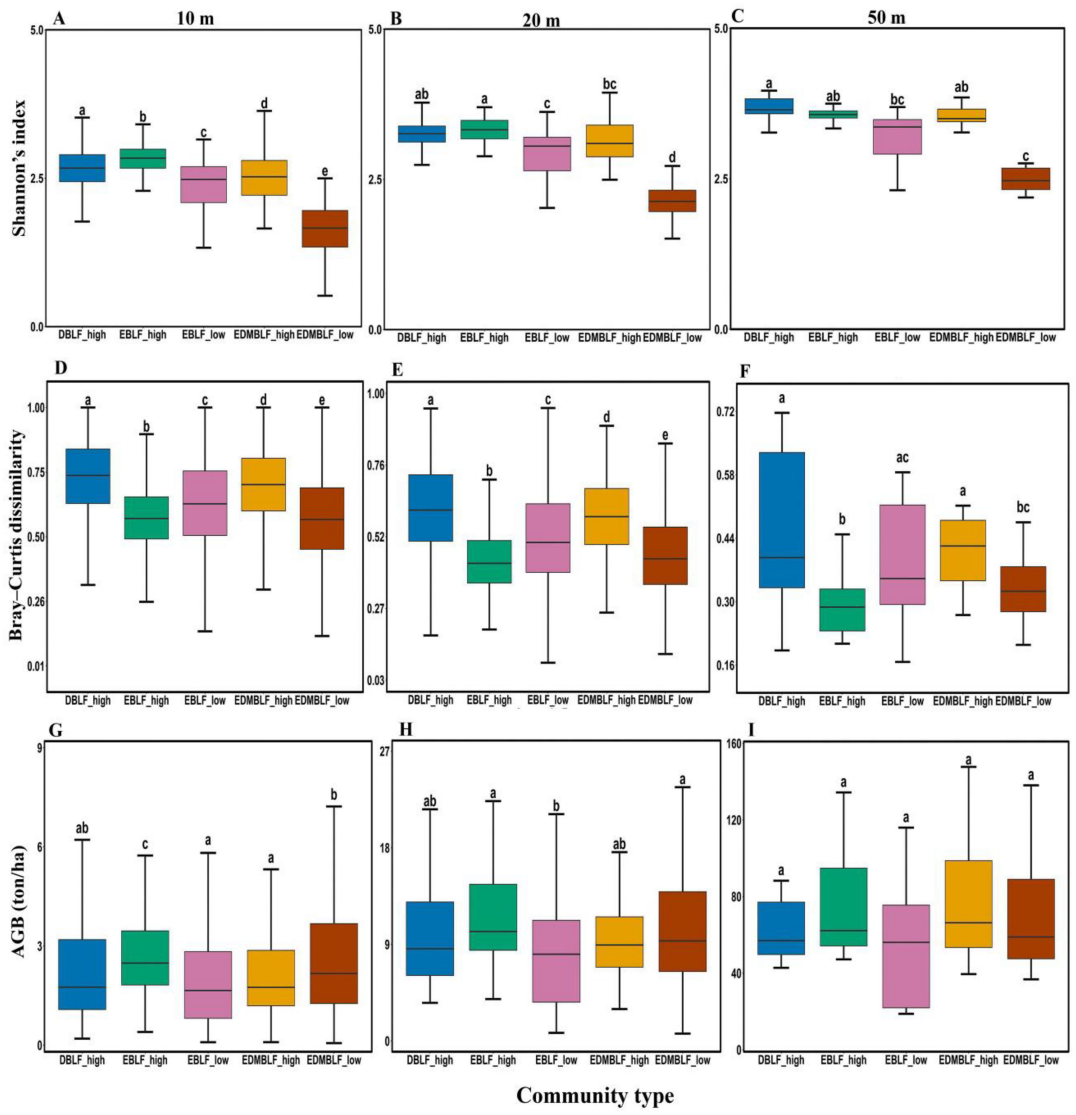
Comparison of alpha diversity (Shannon’s index), beta diversity (Bray–Curtis dissimilarity) and aboveground biomass (AGB) across spatial scales and community types: **(A–C)** Shannon’s index, **(D–F)** Bray–Curtis dissimilarity, and **(G–I)** AGB. Differences among community types were evaluated using Kruskal–Wallis tests followed by Dunn’s *post hoc* pairwise comparisons. Different lowercase letters above boxplots indicate statistically significant differences among community types (*p* ≤ 0.05).

Across spatial scales, clear differences among community types were observed for both Shannon’s index and Bray–Curtis dissimilarity. At the 10 m scale, EBLF_high and DBLF_high generally exhibited higher alpha diversity, whereas EDMBLF_low showed lower median values and reduced variability (**Figures 3A–C**). For beta diversity, DBLF_high and EDMBLF_high displayed higher Bray–Curtis dissimilarity, whereas EBLF_high showed relatively lower compositional variation compared to some community types (**Figures 3D–F**). Both Shannon’s index and Bray–Curtis dissimilarity exhibited decreasing differentiation and more similar distributions among community types with increasing spatial scale, leading to partial convergence at 50 m, although some differences remained.

Community differences in AGB were significant at 10 m and 20 m (*p* < 0.001) but were not significant at 50 m (**Supplementary Tables 2**, **S3**). At the fine spatial scales, differences, including higher median AGB in EBLF_high and EDMBLF_low and lower, more variable values in EBLF_low, became increasingly indistinguishable at broader spatial extents (**Figures 3G–I**). Similarly, spatial variation in biomass (AGB_diff) showed strong scale-dependent differences, with highly significant variation among community types at 10 m and 20 m but no significant differences at 50 m (**Supplementary Figure 3**; **Supplementary Tables 6**, **S7**).

### 3.2 Relationships between alpha diversity and aboveground biomass across different spatial scales and community types

**Figure 4:**
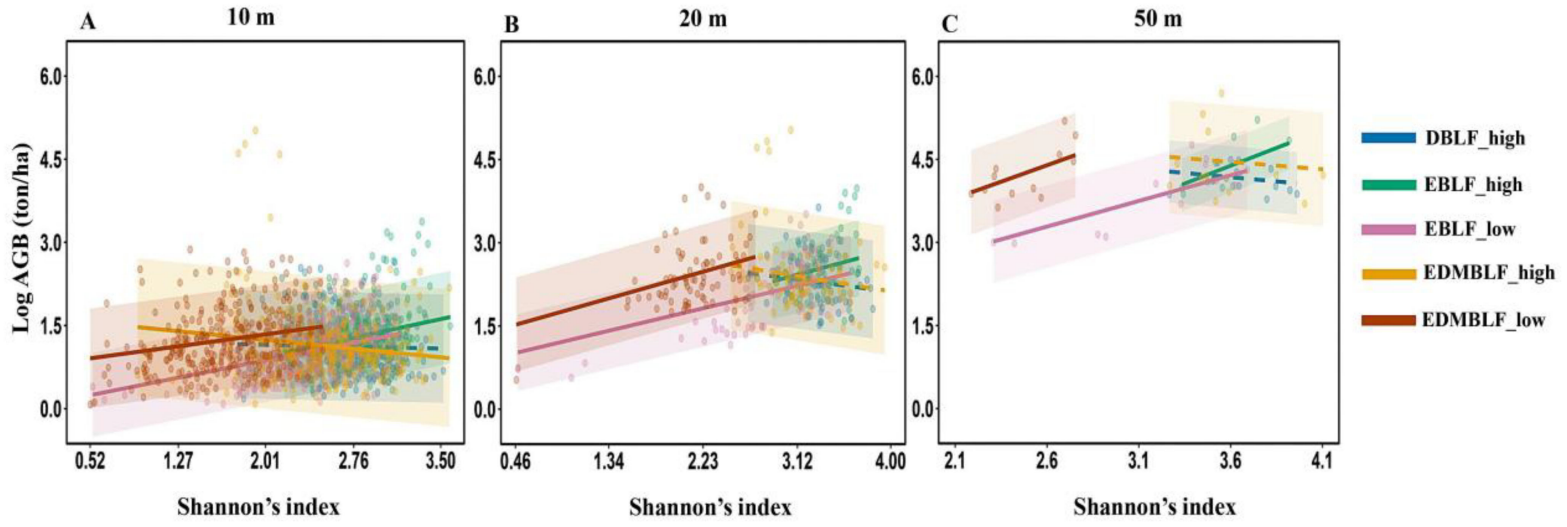
Relationships between alpha diversity (Shannon’s index) and log–transformed aboveground biomass (AGB) across spatial scales and community types analyzed using spatial lag regression models. Panel **(A)** represents Shannon’s index and log AGB at the 10 m scale, Panel **(B)** at the 20 m scale, and Panel **(C)** at the 50 m scale. Points represent individual sampling units. Solid lines indicate significant relationships, whereas dashed lines denote non–significant relationships. Shaded areas represent approximate 95% confidence intervals around fitted model predictions. Community types are distinguished by color (blue: DBLF_high; bluish green: EBLF_high; purple: EBLF_low; orange: EDMBLF_high; dark reddishorange: EDMBLF_low).

At the 10 m scale, significant relationships between Shannon’s index and aboveground biomass (AGB) were observed in most community types, while the direction and strength of these relationships differed among forest communities and across spatial scales (**Figure 4**). Evergreen broad–leaved forests consistently exhibited positive alpha diversity–AGB relationships at all spatial scales (10 m, 20 m, and 50 m), with the relationship becoming stronger at broader scales (**Supplementary Table 8**). In contrast, DBLF_high showed non-significant negative relationships between Shannon’s index and AGB across scales. EDMBLF_high exhibited a negative relationship at each spatial scale, although the relationship was statistically significant only at the 10 m scale. Conversely, EDMBLF_low demonstrated significant positive relationships at all spatial scales. Spatial lag interaction models further showed that the Shannon diversity–AGB relationship did not differ significantly between high- and low-elevation EBLF stands at any spatial scale, whereas significant elevational differences were detected in EDMBLF across all scales (p < 0.001) (**Supplementary Figure 4**).

### 3.3 Relationships between beta diversity and aboveground biomass differences across different spatial scales and community types

**Figure 5:**
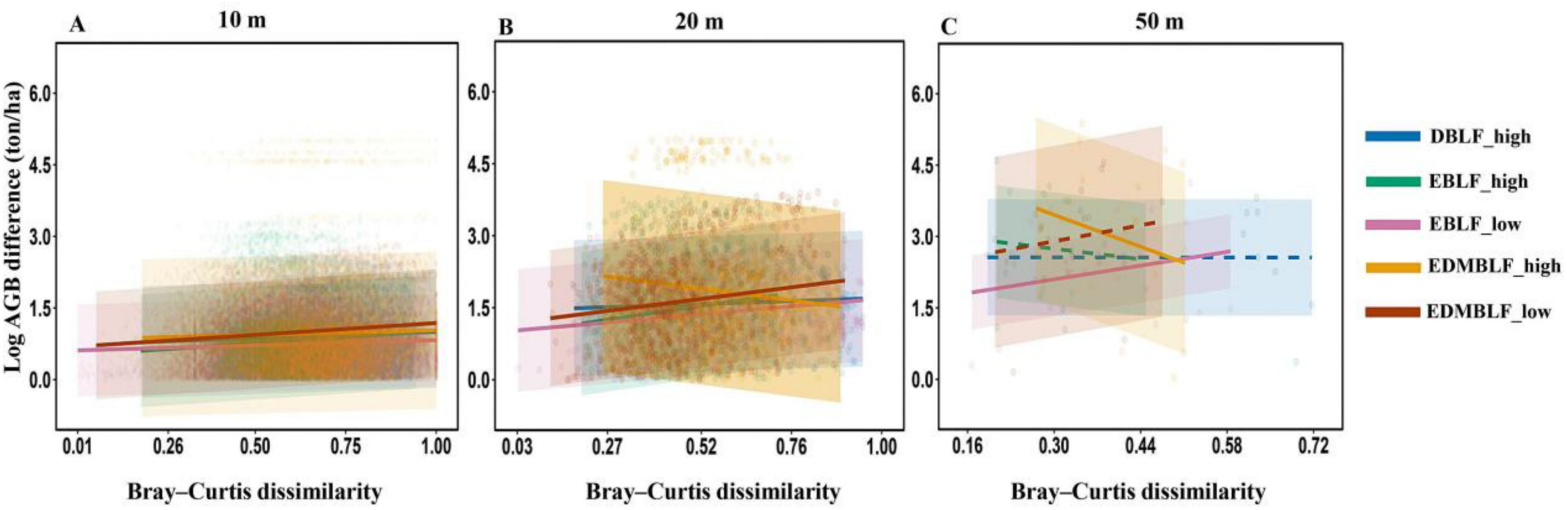
Relationships between beta diversity (Bray–Curtis dissimilarity) and log–transformed aboveground biomass (AGB) difference across spatial scales and community types analyzed using spatial lag regression models. Panel **(A)** represents Bray–Curtis dissimilarity and log AGB difference at the 10 m scale, Panel **(A)** at the 20 m scale, and Panel **(C)** at the 50 m scale. Points represent individual pairwise comparisons among sampling units. Solid lines indicate significant relationships, whereas dashed lines denote non–significant relationships. Shaded areas represent approximate 95% confidence intervals around fitted model predictions. Community types are distinguished by color (blue: DBLF_high; bluish green: EBLF_high; purple: EBLF_low; orange: EDMBLF_high; dark reddish-orange: EDMBLF_low).

AGB_diff was associated with Bray–Curtis dissimilarity across spatial scales, with the strength and significance of these associations varying among community types (**Figure 5**; **Supplementary Table 9**). At the 10 m scale, significant positive relationships were observed in all community types. At the 20 m scale, most community types retained significant positive relationships, whereas EDMBLF_high exhibited a significant negative relationship. At the 50 m scale, DBLF_high and EBLF_high showed non-significant negative relationships, whereas EBLF_low maintained a significant positive relationship at 50 m. EDMBLF_high remained significantly negatively associated with Bray–Curtis dissimilarity at 50 m, while EDMBLF_low exhibited a non-significant positive relationship at the 50 m scale. Significant elevational effects on the relationship between Bray–Curtis dissimilarity and AGB differences were detected in both EBLF and EDMBLF across all spatial scales (*p* < 0.001), indicating that elevation modified beta diversity–biomass relationships (**Supplementary Figure 5**). Residual spatial autocorrelation was largely removed after spatial lag model fitting across community types and spatial scales. However, weak but significant residual autocorrelation persisted in the EBLF_low community at the 50 m scale (**Supplementary Tables 12**, **S13**).

## 4 Discussion

### 4.1 Contrasting elevational responses of diversity–biomass relationships in mixed and evergreen forests

The relationship between alpha diversity and aboveground biomass (AGB) varied across forest types and elevations. Mixed forests showed distinct elevational differences in these relationships. At low elevation, mixed forests consistently exhibited positive diversity–biomass relationships, whereas at high elevation the relationship weakened, with a significant negative association detected at the 10 m scale. A similar but non-significant negative trend was observed in deciduous broad‐leaved forests at high elevation (DBLF_high) (**Figure 4**; **Supplementary Table 8**). Such context-dependent BEF relationships have also been reported in forests experiencing relatively harsh environmental conditions, where environmental filtering may reduce the positive influence of diversity on biomass accumulation (Jucker et al., 2016; Liang et al., 2016; Ratcliffe et al., 2017; van der Plas, 2019; Huang et al., 2018; Barry et al., 2021).

Previous studies have shown that lower temperatures and shorter growing seasons can constrain productivity and biomass accumulation in deciduous forests (Kikuzawa, 1995; Reich, 2014; Körner, 2007). In addition, more open canopy conditions may facilitate the presence of understory species, increasing local diversity without substantially increasing stand biomass. Comparable patterns, in which greater diversity is not associated with higher biomass, have also been reported in forests subject to strong environmental constraints or resource limitation (Grime, 1998; Ratcliffe et al., 2017; van der Plas, 2019).

In evergreen broad‐leaved forests at both low and high elevations, diversity–biomass relationships remained consistently positive (**Figure 4**; **Supplementary Table 8**). These results are consistent with BEF theory, which proposes that species coexistence can promote greater biomass through complementary resource use and vertical canopy stratification (Díaz and Cabido, 2001; Cardinale et al., 2012; Forrester and Bauhus, 2016). The relatively stable positive relationships observed in evergreen forests across elevations may indicate that evergreen species maintain more continuous biomass accumulation under varying environmental conditions than deciduous species. Similar patterns have been documented in subtropical and temperate forests where evergreen‐dominated communities maintained relatively stable productivity across environmental gradients (Paquette and Messier, 2011; Liang et al., 2016). Importantly, the interaction analysis showed no significant difference between high and low elevations in the Shannon–AGB relationship in EBLF (**Supplementary Figures 4A–C**), indicating that this positive relationship was maintained across the elevational gradient.

Overall, the relationship between alpha diversity and AGB supported our hypothesis. Together, these findings support growing evidence that BEF relationships are strongly context-dependent and can differ substantially across forest types and environmental gradients (Liang et al., 2016; Ratcliffe et al., 2017; van der Plas, 2019; Catford et al., 2022).

### 4.2 Beta diversity–biomass relationships vary among forest types and elevations

The relationship between beta diversity (Bray–Curtis dissimilarity) and spatial variation in aboveground biomass (AGB_diff) varied among forest communities and elevations, with both positive and negative associations observed (**Figure 5**; **Supplementary Table 9**). Evergreen and mixed forests differed in the direction and consistency of these relationships, suggesting that the contribution of compositional turnover to biomass variation depends on forest composition and environmental conditions. Similar context-dependent biodiversity–ecosystem functioning relationships have been reported in previous studies across environmental gradients (Loreau et al., 2003; Baselga, 2010; Mori et al., 2018; Isbell et al., 2017).

Evergreen broad‐leaved forests mainly showed positive relationships between beta diversity and AGB_diff across elevations and scales. Whereas, in mixed evergreen–deciduous forests, the relationship between beta diversity and AGB_diff differed between elevations. EDMBLF_low generally maintained positive relationships, and EDMBLF_high shifted toward negative relationships, particularly at broader spatial scales. This pattern suggests that the contribution of compositional turnover to biomass variation may decline under stronger environmental constraints. The weaker relationships observed at high elevation may reflect changes in species composition and environmental conditions that alter the association between species turnover and biomass variation among subplots. Under these conditions, biomass differences may also be influenced by stand structure and tree-size distribution in addition to compositional turnover (Keddy, 1992; Weiher and Keddy, 1995; Enquist et al., 2009).

Differences in species composition between evergreen and deciduous taxa may contribute to variation in stand structure and biomass among subplots. However, environmental stress at higher elevations may reduce the strength and consistency of these biodiversity–biomass relationships (Loreau et al., 2003; Hooper et al., 2005; Kraft et al., 2015; Mori et al., 2018).

In DBLF_high, beta diversity was positively associated with AGB_diff at the 10 m and 20 m scales, but this relationship became non-significant at the 50 m scale (**Figure 5**; **Supplementary Table 9**). This result suggests that compositional turnover alone may not strongly explain biomass variation in this forest type. Instead, biomass differences may reflect multiple ecological factors operating simultaneously, including variation in stand structure and species dominance under cooler high-elevation conditions. Similar patterns have been observed in forest ecosystems where environmental stress weakens biodiversity–ecosystem functioning relationships (Körner, 2007; Sundqvist et al., 2013).

Overall, the relationship between beta diversity and AGB_diff supported our hypothesis (**Figure 1B**). These findings suggest that the influence of compositional turnover on biomass variation is context-dependent and regulated by forest composition and environmental conditions rather than following a single consistent elevational trend (Loreau et al., 2003; Baselga, 2010; Isbell et al., 2017; Mori et al., 2018; van der Plas et al., 2023).

### 4.3 Scale-dependent responses of biodiversity–biomass relationships

The relationships between biodiversity and biomass were influenced by both spatial scale and forest community type, although differences among community types were generally more pronounced than differences among spatial scales. Across both alpha diversity and beta diversity, significant relationships were more frequently observed at the 10 m scale, particularly for beta diversity. Fine spatial grains are more likely to capture local neighborhood interactions, species coexistence processes, and fine-scale environmental heterogeneity, thereby enhancing the detectability of biodiversity effects on biomass (Levin, 1992; Loreau et al., 2003; Huang et al., 2018; Xu et al., 2022).

As spatial grain increased from 10 m to 20 m and 50 m, biodiversity–biomass relationships became increasingly dependent on forest community context. However, the response to spatial scale was not uniform across statistical properties of the relationships. Specifically, while statistical significance generally declined at broader scales, particularly for beta diversity, the absolute magnitude of regression slopes did not show a consistent monotonic decrease, and in several cases increased with spatial scale. This indicates that spatial scale influences the detectability and slope magnitude of biodiversity–biomass relationships in different ways, and these two aspects do not necessarily follow the same directional trend.

Recent BEF theory emphasizes that biodiversity–ecosystem functioning relationships are inherently scale-dependent, as spatial grain influences not only observed effect sizes but also the underlying ecological processes that structure community–function relationships, including environmental heterogeneity, species turnover, and spatial insurance effects (Thompson et al., 2021; Mori et al., 2021; Catford et al., 2022; Isbell et al., 2023). Spatial aggregation may decouple effect size from statistical significance by simultaneously increasing environmental heterogeneity while reducing statistical detectability of biodiversity effects (Mori et al., 2021; Catford et al., 2022).

Accordingly, increasing spatial grain does not uniformly strengthen biodiversity–biomass relationships, despite capturing greater compositional variation and environmental heterogeneity. At broader spatial scales, increasing within-unit heterogeneity and environmental variability may obscure biodiversity effects on biomass, reducing statistical significance even when effect sizes remain present or increase in magnitude. This decoupling is particularly evident in beta diversity, suggesting that compositional turnover may still contribute to biomass variation at larger grains, but with reduced statistical detectability due to aggregation effects and increased environmental noise (Chase and Leibold, 2003; Stein et al., 2014; Mori et al., 2018). Our results suggest that spatial scale reshapes biodiversity–biomass relationships through a dual mechanism affecting both statistical significance and regression slope magnitude, rather than producing a uniform decline in relationship strength.

Overall, these findings partially support the scale-dependent expectations illustrated in **Figure 1C**. While biodiversity–biomass relationships were generally more detectable at finer spatial scales, particularly for beta diversity, the magnitude of regression slopes did not consistently decrease with scale, indicating a more complex scaling pattern than originally assumed. Importantly, forest community context exerted a stronger influence than spatial scale on both the strength and direction of biodiversity–biomass relationships. Supplementary plot-level analyses further support the robustness of these patterns, indicating that the observed scale effects were not driven by individual plot-level influences (**Supplementary Figures 1**, **S2**). Collectively, these results indicate that spatial scale influences biodiversity–biomass relationships, whereas forest community context plays a dominant role in determining their strength and direction.

## 5 Conclusion

Biodiversity–biomass relationships in subtropical forests vary with spatial scale and ecological context. At fine spatial scales, alpha diversity generally shows positive associations with aboveground biomass in evergreen broad-leaved forests and low-elevation mixed forests, whereas relationships in high-elevation mixed forests are weaker and can become negative. Beta diversity is generally positively associated with biomass differences at fine spatial scales across all forest communities. At intermediate scales, relationships remain mostly positive but become more variable among community types. At the largest spatial scale, relationships show strong community-dependent heterogeneity, shifting among positive, negative, and non-significant patterns, indicating a loss of consistency in the turnover–biomass relationship with increasing scale. These results highlight that incorporating spatial scale and forest type is important for interpreting biodiversity–biomass relationships. Maintaining structurally complex and compositionally heterogeneous forests is therefore critical for sustaining productivity and carbon storage across subtropical forest environments.

## Data Availability

The original contributions presented in the study are included in the article/**Supplementary Material**. Further inquiries can be directed to the corresponding author.
