# Supplementary material for: Scale-dependent biodiversity–biomass relationships vary among subtropical forest community types
Source: Front Plant Sci. 2026 Jul 15;17:1869844. doi: 10.3389/fpls.2026.1869844 (PMC13415502; doi:10.3389/fpls.2026.1869844)
Supplement: Supplementary file 1 [file DataSheet1.docx]

**1 Plot-level variation in diversity and biomass across spatial scales** Plot-level assessments revealed notable variability in alpha diversity and AGB across spatial scales and among forest plots (Figure S1). At the 10 m scale, considerable differences were observed, with broad interquartile ranges and frequently non-overlapping medians, highlighting strong heterogeneity at fine spatial scales. This variability decreased with increasing scale, with plots showing more similar distributions at 20 m and converging substantially at 50 m.

Although variability exists within communities, the overall plot-level patterns aligned closely with community-level patterns. Plots belonging to the same community type tended to cluster within similar value ranges, and between-community differences were generally larger than within-community (plot-level) differences, particularly at finer spatial scales. This suggests that community-level differentiation observed in the main analyses reflects consistent trends rather than being driven by individual plots.

Overall, these plot-level comparisons demonstrate that fine-scale heterogeneity is high within forest communities, especially at small spatial extents, but that the scale-dependent patterns identified at the community level remain robust. These findings support using community type as the main analytical focus in this study while recognizing the importance of variability within communities.

**Figure S1** Comparison of alpha diversity (Shannon’s index) and aboveground biomass (AGB) across spatial scales and forest plots: **(A-C)** Shannon’s index, **(D-F)** AGB. Differences among forest plots were evaluated using Kruskal-Wallis tests followed by Dunn’s post hoc pairwise comparisons. Different lowercase letters above the boxplots indicate statistically significant differences among plots (p ≤ 0.05). Colors represent community types (blue: DBLF_high; bluish green: EBLF_high; purple: EBLF_low; orange: EDMBLF_high; dark reddish-orange: EDMBLF_low), with three plots per community. This figure illustrates plot-level heterogeneity and complements the community-level patterns presented in the main text.

Figure S2 Comparison of beta diversity (Bray-Curtis dissimilarity) and aboveground biomass (AGB) difference across spatial scales and forest plots: **(A-C)** Bray-Curtis dissimilarity, **(D-F)** AGB_diff. Differences among forest plots were evaluated using Kruskal-Wallis tests followed by Dunn’s post hoc pairwise comparisons. Different lowercase letters above the boxplots indicate statistically significant differences among plots (*p* ≤ 0.05). Colors represent community types (blue: DBLF_high; bluish green: EBLF_high; purple: EBLF_low; orange: EDMBLF_high; dark reddish-orange: EDMBLF_low), with three plots per community. This figure illustrates plot-level heterogeneity and complements the community-level patterns presented in the main text.

Figure S3 Comparison of aboveground biomass (AGB_diff) difference across spatial scales and community types. Differences among community types were evaluated using Kruskal-Wallis tests followed by Dunn’s post hoc pairwise comparisons. Different lowercase letters above boxplots indicate statistically significant differences among community types (*p* ≤ 0.05).

Figure S4. Effects of elevation on the relationship between alpha diversity (Shannon’s index) and aboveground biomass (AGB) across spatial scales and forest types. Points represent standardized regression coefficients (β), and error bars indicate 95% confidence intervals estimated from spatial lag (SAR) interaction models. Panels **(A–C)** show results for evergreen broad-leaved forests (EBLF), and panels **(D–F)** show results for evergreen-deciduous mixed broad-leaved forests (EDMBLF). The p-values indicate the significance of the Shannon’s index × elevation interaction term, testing whether the alpha diversity–AGB relationship differs between elevations.

Figure S5. Effects of elevation on the relationship between beta diversity (Bray–Curtis dissimilarity) and aboveground biomass differences (AGB_diff) across spatial scales and forest types. Points represent standardized regression coefficients (β), and error bars indicate 95% confidence intervals estimated from spatial lag (SAR) interaction models. Panels **(A–C)** show results for evergreen broad-leaved forests (EBLF), and panels **(D–F)** show results for evergreen-deciduous mixed broad-leaved forests (EDMBLF). The p–values indicate the significance of the Bray–Curtis dissimilarity × elevation interaction term, testing whether the beta diversity–AGB_diff relationship differs between elevations.

**Table S1 The plot information of the study areas.**

| **No.** | **Plot name** | **Longitude WE (°)** | **Latitude NE (°)** | **Elevation (m)** | **Community types** |
| --- | --- | --- | --- | --- | --- |
| 1 | Plot 1 | 119.00702 | 29.65241 | 342 | Evergreen broad–leaved forest |
| 2 | Plot 2 | 118.076 | 29.148 | 683 |  |
| 3 | Plot 3 | 118.89073 | 28.38134 | 747 |  |
| 4 | Plot 4 | 122.32441 | 29.8322 | 61 | Evergreen and deciduous mixed broad–leaved forest |
| 5 | Plot 5 | 122.26578 | 29.8183 | 146 |  |
| 6 | Plot 6 | 122.26937 | 29.8139 | 222 |  |
| 7 | Plot 7 | 119.06734 | 27.54654 | 808 | Evergreen broad–leaved forest |
| 8 | Plot 8 | 119.67048 | 27.70563 | 960 |  |
| 9 | Plot 9 | 119.06993 | 27.54993 | 967 |  |
| 10 | Plot 10 | 118.89317 | 30.10737 | 975 | Evergreen and deciduous mixed broad–leaved forest |
| 11 | Plot 11 | 118.12417 | 29.27722 | 1004 |  |
| 12 | Plot 12 | 119.42947 | 30.339 | 1069 |  |
| 13 | Plot 13 | 118.91028 | 30.11396 | 926 | Deciduous broad–leaved forest |
| 14 | Plot 14 | 118.89179 | 28.36359 | 971 |  |
| 15 | Plot 15 | 118.7667 | 29.24547 | 1050 |  |

**Table S2 Kruskal-Wallis test results for alpha diversity (Shannon’s index) and AGB across different spatial scales and community types.**

| **Index** | **Spatial scale** | **Chi-square value** | **p-value** |
| --- | --- | --- | --- |
| Shannon’s index | 10 m | 731.673 | 4.827e-157*** |
|  | 20 m | 201.593 | 1.708e-42*** |
|  | 50 m | 34.358 | 6.291e-07*** |
| AGB | 10 m | 67.306 | 8.405e-14*** |
|  | 20 m | 19.291 | 6.891e-04*** |
|  | 50 m | 3.368 | 0.498 |

**Table S3 Post-hoc Dunn’s test adjusted p-values for alpha diversity (Shannon’s index) and AGB across different spatial scales and community types.**

| **Shannon’s index**  **comparison** | **10 m** | **20 m** | **50 m** |
| --- | --- | --- | --- |
| DBLF_high-EBLF_high | 8.707e-07*** | 1.000 | 1.000 |
| DBLF_high-EBLF_low | 1.399e-11*** | 7.552e-06*** | 0.017* |
| EBLF_high-EBLF_low | 1.334e33*** | 7.222e-09*** | 0.185 |
| DBLF_high-EDMBLF_high | 2.233e-04*** | 0.111 | 1.000 |
| EBLF_high-EDMBLF_high | 6.314e-20*** | 0.002** | 1.000 |
| EBLF_low-EDMBLF_high | 0.018* | 0.059 | 0.451 |
| DBLF_high-EDMBLF_low | 3.137e-90*** | 1.465e-29*** | 8.724e-07*** |
| EBLF_high-EDMBLF_low | 3.812e-142*** | 4.254e-36*** | 5.850e-05*** |
| EBLF_low-EDMBLF_low | 2.772e-39*** | 1.783e-10*** | 0.108 |
| EDMBLF_high-EDMBLF_low | 5.081e-58*** | 3.061e-19*** | 3.282e-04*** |
| **AGB comparison** | **10 m** | **20 m** | **50 m** |
| DBLF_high-EBLF_high | 9.139e-07*** | 0.062 | 1.000 |
| DBLF_high-EBLF_low | 0.086 | 0.365 | 1.000 |
| EBLF_high-EBLF_low | 1.478e-13*** | 8.735e-05*** | 0.614 |
| DBLF_high-EDMBLF_high | 1.000 | 1.000 | 1.000 |
| EBLF_high-EDMBLF_high | 1.677e-07*** | 0.178 | 1.000 |
| EBLF_low-EDMBLF_high | 0.188 | 0.141 | 0.587 |
| DBLF_high-EDMBLF_low | 0.104 | 1.000 | 1.000 |
| EBLF_high-EDMBLF_low | 0.019* | 0.554 | 1.000 |
| EBLF_low-EDMBLF_low | 1.317e-05*** | 0.035* | 1.000 |
| EDMBLF_high-EDMBLF_low | 0.044* | 1.000 | 1.000 |

**Table S4 Kruskal-Wallis test results for beta diversity (Bray-Curtis** **dissimilarity) across different spatial scales and community types.**

| **Index** | **Spatial scale** | **Chi-square value** | **p-value** |
| --- | --- | --- | --- |
| **Bray-Curtis** **dissimilarity** | 10 m | 22473.527 | < 0.001*** |
|  | 20 m | 1858.908 | < 0.001*** |
|  | 50 m | 45.662 | < 0.001*** |

**Table S5 Post-hoc Dunn’s test adjusted p-values for beta diversity (Bray-Curtis** **dissimilarity) across different spatial scales and community types.**

| **Bray-Curtis dissimilarity comparison** | **10 m** | **20 m** | **50 m** |
| --- | --- | --- | --- |
| DBLF_high-EBLF_high | < 0.001*** | 4.547e-261*** | 1.460e-07*** |
| DBLF_high-EBLF_low | < 0.001*** | 1.763e-74*** | 0.149 |
| EBLF_high-EBLF_low | < 0.001*** | 1.925e-58*** | 0.004** |
| DBLF_high-EDMBLF_high | 7.645e-96*** | 0.0011** | 1.000 |
| EBLF_high-EDMBLF_high | < 0.001*** | 1.913e-208*** | 3.846e-07*** |
| EBLF_low-EDMBLF_high | < 0.001*** | 7.169e-48*** | 0.228 |
| DBLF_high-EDMBLF_low | < 0.001*** | 3.696e-189*** | 5.389e-04 *** |
| EBLF_high-EDMBLF_low | 1.024e-07 *** | 1.151e-06 *** | 0.471 |
| EBLF_low-EDMBLF_low | < 0.001*** | 1.179e-27 *** | 0.445 |
| EDMBLF_high-EDMBLF_low | < 0.001*** | 7.707e-145 *** | < 0.001*** |

**Table S6 Kruskal-Wallis test results for aboveground biomass differences across different spatial scales and community types.**

| **Index** | **Spatial scale** | **Chi-square value** | **p-value** |
| --- | --- | --- | --- |
| **AGB_diff** | 10 m | 2175.830 | < 0.001*** |
|  | 20 m | 167.948 | 2.883e-35*** |
|  | 50 m | 6.064 | 0.194 |

**Table S7 Post-hoc Dunn’s test adjusted p-values for aboveground biomass differences across different spatial scales and community types.**

| **AGB_diff comparison** | **10 m** | **20 m** | **50 m** |
| --- | --- | --- | --- |
| DBLF_high-EBLF_high | 1.029e-81*** | 1.862e-11*** | 1.000 |
| DBLF_high-EBLF_low | 4.980e-186*** | 7.119e-19*** | 1.000 |
| EBLF_high-EBLF_low | 1.819e-22*** | 0.177 | 1.000 |
| DBLF_high-EDMBLF_high | 8.252e-12*** | 1.000 | 0.804 |
| EBLF_high-EDMBLF_high | 2.266e-33*** | 1.085e-13*** | 0.567 |
| EBLF_low-EDMBLF_high | 2.316e-107*** | 9.849e-22*** | 0.264 |
| DBLF_high-EDMBLF_low | 2.853e-39*** | 1.000 | 1.000 |
| EBLF_high-EDMBLF_low | 1.784e-230*** | 1.537e-11*** | 0.818 |
| EBLF_low-EDMBLF_low | < 0.001*** | 5.554e-19*** | 0.404 |
| EDMBLF_high-EDMBLF_low | 7.3244e-91*** | 1.000 | 1.000 |

**Table S8 Spatial lag model regression results for alpha diversity and aboveground biomass across different spatial scales and community types.**

|  |  | **AGB ~ Shannon’s index** | | | |  |
| --- | --- | --- | --- | --- | --- | --- |
| **Community type** | **Spatial scale** | **Fixed effect** | | **p-value** | **ρ (Rho)** | **R² (pseudo, correlation-based)** |
|  |  | **Intercept** | **Slope** |  |  |  |
| DBLF_high | 10 m | -0.002 | -0.029 | 0.602 | 0.192 | 0.038 |
|  | 20 m | -0.002 | -0.148 | 0.193 | 0.147 | 0.045 |
|  | 50 m | 0.081 | -0.239 | 0.378 | 0.306 | 0.124 |
| EBLF_high | 10 m | 0.009 | 0.225 | 1.535e-05*** | 0.385 | 0.219 |
|  | 20 m | 0.032 | 0.211 | 0.015* | 0.607 | 0.478 |
|  | 50 m | 0.067 | 0.475 | 9.362e-4*** | 0.696 | 0.778 |
| EBLF_low | 10 m | -0.002 | 0.347 | 2.288e-12*** | 0.454 | 0.456 |
|  | 20 m | 0.011 | 0.429 | 4.338e-08*** | 0.542 | 0.714 |
|  | 50 m | -0.026 | 0.663 | 2.182e-4*** | 0.375 | 0.752 |
| EDMBLF_high | 10 m | -7.948e-4 | -0.142 | 0.014* | -0.056 | 0.020 |
|  | 20 m | -0.025 | -0.148 | 0.130 | 0.521 | 0.328 |
|  | 50 m | 0.118 | -0.111 | 0.581 | 0.701 | 0.551 |
| EDMBLF_low | 10 m | 0.003 | 0.212 | 1.291e-05*** | 0.530 | 0.358 |
|  | 20 m | 0.033 | 0.327 | 3.336e-4*** | 0.568 | 0.454 |
|  | 50 m | 0.036 | 0.500 | 0.024* | 0.380 | 0.543 |

**Table S9 Spatial lag model regression results for beta diversity and aboveground biomass differences across different spatial scales and community types.**

| **AGB_diff ~ Bray-Curtis dissimilarity** | | | | | | |
| --- | --- | --- | --- | --- | --- | --- |
| **Community type** | **Spatial scale** | **Fixed effect** | | **p-value** | **ρ (Rho)** | **R² (pseudo, correlation-based)** |
|  |  | **Intercept** | **Slope** |  |  |  |
| DBLF_high | 10 m | 0.014 | 0.062 | < 0.001*** | 0.273 | 0.039 |
|  | 20 m | -0.008 | 0.051 | 0.034* | 0.405 | 0.129 |
|  | 50 m | -0.001 | -0.054 | 0.697 | 0.713 | 0.353 |
| EBLF_high | 10 m | -0.082 | 0.088 | < 0.001*** | 0.457 | 0.194 |
|  | 20 m | -0.044 | 0.129 | 7.209e-11*** | 0.532 | 0.311 |
|  | 50 m | 0.027 | -0.150 | 0.187 | 0.803 | 0.562 |
| EBLF_low | 10 m | -0.047 | 0.067 | < 0.001*** | 0.530 | 0.209 |
|  | 20 m | -0.016 | 0.152 | 8.66e-15*** | 0.570 | 0.329 |
|  | 50 m | -1.823e-16 | 0.287 | 1.891e-4*** | 0.871 | 0.810 |
| EDMBLF_high | 10 m | 0.016 | 0.032 | 2.047e-08*** | 0.299 | 0.041 |
|  | 20 m | 0.031 | -0.109 | 1.159e-07*** | 0.527 | 0.264 |
|  | 50 m | 0.004 | -0.242 | 0.023* | 0.815 | 0.610 |
| EDMBLF_low | 10 m | -0.029 | 0.133 | < 0.001*** | 0.493 | 0.187 |
|  | 20 m | -0.033 | 0.157 | 2.22e-16*** | 0.577 | 0.386 |
|  | 50 m | 0.013 | 0.166 | 0.259 | 0.572 | 0.234 |

**Table S10 Moran's I statistics for spatial autocorrelation of alpha diversity and aboveground biomass across community types and spatial scales.**

| **Community type** | **Spatial scale** | **Moran’s I**  **(Shannon)** | **p-value** | **Moran’s I (AGB)** | **p-value** |
| --- | --- | --- | --- | --- | --- |
| DBLF_high | 10 m | 0.167 | 7.535e-07*** | 0.037 | 0.128 |
|  | 20 m | 0.214 | 4.763e-4*** | 0.066 | 0.124 |
|  | 50 m | 0.003 | 0.231 | -0.025 | 0.312 |
| EBLF_high | 10 m | -0.023 | 0.708 | 0.015 | 0.305 |
|  | 20 m | -0.061 | 0.752 | -0.025 | 0.570 |
|  | 50 m | -0.055 | 0.386 | -0.185 | 0.771 |
| EBLF_low | 10 m | -0.077 | 0.980 | -0.044 | 0.867 |
|  | 20 m | -0.022 | 0.552 | -0.066 | 0.775 |
|  | 50 m | -0.184 | 0.768 | -0.186 | 0.762 |
| EDMBLF_high | 10 m | 0.023 | 0.232 | 0.023 | 0.223 |
|  | 20 m | 0.054 | 0.165 | -0.110 | 0.928 |
|  | 50 m | -0.064 | 0.416 | -0.226 | 0.858 |
| EDMBLF_low | 10 m | -0.072 | 0.973 | -0.093 | 0.994 |
|  | 20 m | -0.077 | 0.832 | -0.070 | 0.796 |
|  | 50 m | -0.164 | 0.708 | -0.247 | 0.889 |

**Table S11 Moran's I statistics for spatial autocorrelation of beta diversity and aboveground biomass differences across community types and spatial scales.**

| **Community type** | **Spatial scale** | **Moran’s I**  **(Bray_Curtis)** | **p-value** | **Moran’s I (AGB_diff)** | **p-value** |
| --- | --- | --- | --- | --- | --- |
| DBLF_high | 10 m | 0.079 | < 0.001*** | 0.136 | < 0.001*** |
|  | 20 m | 0.017 | 0.007** | 0.032 | 3.547e-06*** |
|  | 50 m | -0.080 | 0.861 | -0.064 | 0.780 |
| EBLF_high | 10 m | 0.046 | < 0.001*** | 0.027 | 6.538e-59*** |
|  | 20 m | 0.074 | 4.041e-25*** | 0.058 | 4.108e-16*** |
|  | 50 m | -0.091 | 0.915 | -0.062 | 0.762 |
| EBLF_low | 10 m | 0.157 | < 0.001*** | 0.012 | 9.047e-13*** |
|  | 20 m | 0.294 | < 0.001*** | 0.023 | 0.001*** |
|  | 50 m | -0.051 | 0.687 | -0.053 | 0.697 |
| EDMBLF_high | 10 m | 0.097 | < 0.001*** | 0.058 | < 0.001*** |
|  | 20 m | 0.059 | 7.694e-17*** | -0.002 | 0.574 |
|  | 50 m | 0.079 | 0.010** | -0.076 | 0.844 |
| EDMBLF_low | 10 m | 0.043 | < 0.001*** | 0.041 | < 0.001*** |
|  | 20 m | 0.043 | 1.080e-09*** | 0.021 | 0.002** |
|  | 50 m | -0.023 | 0.448 | -0.085 | 0.889 |

**Table S12 Residual spatial autocorrelation (Moran’s I) of spatial lag models relating alpha diversity and aboveground biomass.**

| **Community type** | **Spatial scale** | **Moran’s I**  **(Residual)** | **p-value** |
| --- | --- | --- | --- |
| DBLF_high | 10 m | -0.018 | 0.642 |
|  | 20 m | -0.020 | 0.536 |
|  | 50 m | 0.024 | 0.224 |
| EBLF_high | 10 m | -0.061 | 0.929 |
|  | 20 m | -0.088 | 0.842 |
|  | 50 m | -0.035 | 0.343 |
| EBLF_low | 10 m | -0.056 | 0.904 |
|  | 20 m | 0.036 | 0.256 |
|  | 50 m | -0.084 | 0.481 |
| EDMBLF_high | 10 m | -0.004 | 0.508 |
|  | 20 m | -0.001 | 0.435 |
|  | 50 m | 0.028 | 0.196 |
| EDMBLF_low | 10 m | -0.045 | 0.851 |
|  | 20 m | 0.045 | 0.219 |
|  | 50 m | -0.166 | 0.692 |

**Table S13 Residual spatial autocorrelation (Moran’s I) of spatial lag models relating beta diversity and aboveground biomass differences.**

| **Community type** | **Spatial scale** | **Moran’s I**  **(Residual)** | **p-value** |
| --- | --- | --- | --- |
| DBLF_high | 10 m | -0.002 | 0.723 |
|  | 20 m | -0.001 | 0.507 |
|  | 50 m | 0.033 | 0.148 |
| EBLF_high | 10 m | -0.014 | 0.910 |
|  | 20 m | -0.002 | 0.531 |
|  | 50 m | 0.023 | 0.190 |
| EBLF_low | 10 m | -0.014 | 0.910 |
|  | 20 m | 0.008 | 0.270 |
|  | 50 m | 0.070 | 0.048* |
| EDMBLF_high | 10 m | -0.003 | 0.798 |
|  | 20 m | -0.002 | 0.530 |
|  | 50 m | 0.005 | 0.288 |
| EDMBLF_low | 10 m | -0.028 | 1.000 |
|  | 20 m | -0.006 | 0.638 |
|  | 50 m | -0.005 | 0.343 |
